# Effectiveness of preoperative tranexamic acid in reducing blood loss during caesarean section at Aminu Kano Teaching Hospital, Kano: a randomized controlled trial

**DOI:** 10.11604/pamj.2021.39.34.21938

**Published:** 2021-05-12

**Authors:** Rasaki Olaiya Oseni, Mohammad Zakari, Natalia Adamou, Usman Aliyu Umar

**Affiliations:** 1 Department of Obstetrics and Gynaecology, Aminu Kano Teaching Hospital, Kano, Nigeria,; 2 Department of Obstetrics and Gynaecology, Aminu Kano Teaching Hospital, Bayero University, Kano, Nigeria

**Keywords:** Tranexamic acid, caesarean section, haemorrhage

## Abstract

**Introduction:**

bleeding during and after caesarean section is one of the contributors to maternal mortality and morbidity. Tranexamic acid can be given before surgery to significantly reduce the amount of blood loss during caesarean section. The objective was to evaluate the effectiveness of preoperative tranexamic acid in reducing blood loss during caesarean section at Aminu Kano Teaching Hospital, Kano.

**Methods:**

this was a randomized double blind placebo controlled study that was carried out among 244 women who were to have emergency caesarean section between December 2017 and June 2018 and were randomly assigned to the study group or control group. Women in the study group received lg (10mls) of tranexamic acid intravenously while women in the control group received 10ml of normal saline. Oxytocin was administered in the two groups according to protocol. Measurement of blood loss was done immediately after surgery. Postoperative drop in haemoglobin and haematocrit were also determined. Statistical analysis was done using SPSS Version 22.

**Results:**

the average intra operative blood loss was 414.0 ml in the study group and 773.8 ml in the control group (t = - 16.18, p ≤ 0.01). Average postoperative haemoglobin was 10.1 g/dl in the study group and 9.5 g/dl in the control group (t = 4.99, p ≤ 0.01). Average postoperative haematocrit was 31.5% in the study group and 29.9% in the control group (t = 4.70, p ≤ 0.01).

**Conclusion:**

there was a significant reduction in the blood loss when preoperative tranexamic acid was given to patients who were to undergo emergency caesarean section.

## Introduction

Caesarean section (CS) is the commonest indication for surgery in Sub-Saharan Africa [1]. Although, it has evolved to become a necessity in improving obstetric care [2], it is not without complications due to the strong relationship between increase blood loss and mortality [3, 4]. Women whose labors eventually end with an emergency caesarean, face much higher risks of severe postpartum haemorrhage [5]. Associated with haemorrhage would be the need for blood transfusion and its inherent hazards [6].

Postpartum haemorrhage (PPH) is the main cause of maternal mortality worldwide, therefore, interventions towards its prevention need to be prioritized [7]. Worldwide, massive obstetric haemorrhage is responsible for 25% of the estimated 358,000 maternal deaths each year [8, 9]. More than 80% of reported haemorrhage deaths were classified as PPH [10]. The proportions of maternal deaths attributable to PPH vary considerably between developed and developing countries [7]. PPH accounts for 8% of maternal deaths in developed regions [11] while it accounts for 53% of maternal deaths in the Philippines and 43% in Indonesia [12]. It is responsible for 32% of maternal deaths in Northern Africa [11]. Bleeding during and after caesarean section accounts for 26.2% of maternal deaths due to obstetric haemorrhage in South Africa [13].

Nigeria and India are estimated to account for over one third of all maternal deaths worldwide in 2015 with Nigeria accounting for approximately 19% maternal deaths [14]. Official estimates attribute 23% of the maternal mortality burden in Nigeria to PPH [15]. A Teaching Hospital in Ebonyi reported 28.5% of maternal deaths following caesarean delivery and haemorrhage accounting for 23% of maternal mortality [16]. In Enugu State, a Teaching Hospital also reported obstetric haemorrhage accounting for 23.7% [17] maternal deaths while anaemia was the commonest post caesarean morbidity in 32.5% of women [18]. A Tertiary Health Institution in Sokoto however reported haemorrhage (59.7%) as the commonest complication following caesarean delivery while anaemia was 10.5% [19]. In Aminu Kano Teaching Hospital, postpartum haemorrhage was responsible for 4.5% [20] of the complications following caesarean delivery while postpartum anaemia was as high as 21.3% [20] following caesarean delivery.

Tranexamic acid was found to be effective in reducing blood transfusion requirements in anaemic parturient undergoing LSCS [21]. It is one of the most effective antifibrinolytics. It causes reversible and competitive blockade of the lysine binding sites in plasminogen molecules [22]. Tranexamic acid is Food and Drug Administration (FDA) Pregnancy Category B Drug [22]. It has a good safety profile in LSCS, achieving less drop in postoperative haemoglobin and haematocrit [21]. On cost analysis, TXA use was found to be much more cost-effective in comparison to blood transfusion [21]. Furthermore, the prevalence of anaemia in pregnancy in Kano was found to be 17.5% [23]. Another study in Kano reported a high rate of 24.5% [24] while it was up to 48.1% [25] in Primary Health Care Facilities (PHC) in Kano. Consequently, a postpartum blood loss which is likely to be well tolerated in women without pre-existing anaemia with a larger build could have major implications in pregnant women with anaemia and/or short stature thus the need to reduce blood loss during CS as much as possible. Tranexamic acid (TXA) is a suitable drug for this purpose. It has been given prior to elective caesarean section with significant reduction in blood loss during surgery and at 2 hours postpartum [26]. The complications following CS in our center is found to include postpartum haemorrhage at a rate of 4.5%, postpartum anaemia at a rate of 21.3% [20]. Therefore, the purpose of this study is to examine the effectiveness of preoperative tranexamic acid in reducing blood loss during caesarean section.

## Methods

The study period was from December 2017 to June 2018. Two hundred and forty-four patients who had emergency caesarean section were recruited and randomized into the study group and the control group with 122 patients in each group. Each patient was followed up postoperatively, till discharge from the hospital and the data was analyzed.

**Study design:** it was a randomized double blind placebo controlled study.

**Inclusion criteria:** patients in the age group > 18 years to < 40 years; of low parity (1-4), who were pregnant with singleton, live fetus at gestational age > 37 weeks but < 42 weeks; who were to have primary emergency caesarean section under spinal anaesthesia. They would had been well informed and had consented to participate in the study in the obstetric unit of Aminu Kano Teaching Hospital (AKTH).

**Exclusion criteria:** 1) Non consenting patients. 2) Patients with known allergy to tranexamic acid (TXA). 3) Patients with bleeding disorders. 4) Patients with antepartum haemorrhage. 5) Patients with major hepatic, major cardiac, major renal, major respiratory disorders in pregnancy were excluded. 6) Pregnant women who required prophylaxis and/or treatment for deep vein thrombosis.

**Sample size determination:** the sample size for each group was determined using the statistical formula for comparison, of proportions as follows:

$$n=\frac{1}{\left(1-f\right)}\times \frac{2\times {\left(Z_{\alpha }+Z_{\beta }\right)}^{2}\times P\times (1-P)}{{\left(P_{0}-P_{1}\right)}^{2}}$$

Where: n = minimum sample size; Po = the proportion of participants in the control group that was expected to develop postpartum blood loss of > 400ml without receiving tranexamic acid. A study by Yehia and colleagues in Egypt noted this to be 32.38% from Cochrane systematic review [27]. P1 = the proportion of participants in the experimental group was expected to develop postpartum blood loss >400ml. For this study, a reduction in the risk of postpartum blood loss by 50% was considered statistically significant. P1 was therefore half of 32.38% which was 16.19%, i.e. 0.162. Z_a = was determined from a statistical table based on the value of the level of significance. For this study, it was set at 0.05. Therefore, Z_a = 1.96. Zß = was determined from a statistical table based on the acceptable power of comparison between 2 groups. For this study, power of 80% (0.80) was used, therefore Zß = 0.84. f = proportion of study participants who were dropped from the study eventually due to unforeseen intra-operative events like failure of spinal anaesthesia with conversion to general anaesthesia, intra operative blood transfusion, hysterectomy and patients who ultimately require continuous oxytocin infusion. For this study f = 10% (0.1)

$$P=\frac{P_{0}+P_{1}}{2}$$

= 0.243

Therefore, the minimum sample size that was required for each group to be statistically significant was:

n = 1/ (1-0.1) x 2 x (1.96 + 0.84)^2^ x 0.243 x (1-0.243)/ (0.324 - 0.162)^2^

= 122 subjects per group approximately. A total of 244 subjects was required to make the results statistically significant.

**Patient selection:** patients who were selected for randomization were fully informed about the study. This was done while the patients were being prepared for the operation. To reduce the time needed to obtain the informed consent after decision for emergency operation has been made, potential patients for emergency caesarean section in labour (such as primigravidas; those with slow progress of labour or those on augmentation of labour) were informed about the study on admission by the researcher or the research assistant, should the need arise for an emergency operation. The questionnaire was administered preoperatively where time permitted or postoperatively to obtain information about the patient´s general characteristics and labour details.

**Sampling technique:** simple random sampling was used in this study to assign subjects to either of the two groups. A structured questionnaire was administered to obtain information about the personal data and other relevant information about the study participants. Qualified participants who had given informed consent to participate in the study were randomly allocated into groups. Group A was the study group and group B was the control group.

**Data collection techniques and tools:** the enrolled patients randomly received intravenous (IV) tranexamic acid (Group A) or intravenous normal saline (Group B). All the ampoules of tranexamic acid used throughout the study were produced by the same pharmaceutical company. Study group (Group A) comprised of 122 patients. Each patient received lg (10ml) of tranexamic acid (a clear, colorless solution) withdrawn into a 10ml syringe and given bolus intravenously over 5-10 minutes, at least 5 minutes before commencement of the operation. Oxytocin 10 IU was administered after the delivery of the neonate according to protocol. Control group (Group B) also comprised of 122 patients. Each patient received 10ml of normal saline, given bolus intravenously over 5-10 minutes, at least 5 minutes before commencement of the operation. Oxytocin 10 IU was administered after the delivery of the neonate.

**Measurement of blood loss at surgery:** all materials such as gauze, pads and drapes were weighed with an electronic weighing scale (Ozeri® Epicurean digital kitchen scale, Model: ZK17) before and at the end of surgery. Immediately skin incision was given, blood was soaked by pre-weighed gauze, pads and drapes. On entering the amniotic cavity, amniotic fluid was sucked up by vacuum into a suction bottle. After removal of the placenta, bleeding was soaked by pre-weighed gauze, pads, drapes and/or sucked up by vacuum into a second suction bottle. The volume of blood in the second suction bottle in addition to weight of blood soaked gauze, pads and drapes were considered for measurement of blood loss in this study. The total intra-operative blood loss (ml) = (weight of blood soaked materials during LSCS - weight of dry materials prior to LSCS) + (the volume of blood in the suction bottle after placental delivery in ml). One gram (lg) increase in the weight of blood soaked surgical gauze is equivalent to one milliliter (1ml) of blood lost [28].

**Postoperative monitoring:** all patients were managed according to the postoperative protocol for caesarean section practiced in our department. On the 2^nd^ postoperative day, the researcher or research assistants determined the patient´s haemoglobin (Hb) concentration and packed cell volume (PCV). Patients with Hb value of > 8g/dl were given oral haematinics and they were discharged on the 5^th^ postoperative day once the wound had healed well. Patients with PCV < 8g/dl remained on admission during the period of blood transfusion.

### Definitions

**Postpartum haemorrhage:** postpartum haemorrhage was defined as blood loss in excess of 1 litre in a caesarean section.

**Postpartum anaemia:** this was defined as haemoglobin concentration (Hb) < 10g/dl in postpartum period.

**Length of hospital stay:** this was counted in days with the surgery day being day zero to the day of discharge.

### Data analysis

All data collected from the study were entered into the Statistical Package for Social Sciences (SPSS) computer software version 22.0 for windows. Categorical data was analysed by chi-square test and continuous data was analysed using independent sample T test. The results were expressed as Odds Ratio (Adjusted Odds Ratio) and 95% Confidence Interval for categorical variables and as means and standard deviations for continuous variables. Results were presented in tables. P-value of less than 0.05 was determined to be statistically significant. Ethical clearance was obtained for the study from the Research Ethics committee of Aminu Kano Teaching Hospital, Kano.

## Results

Table 1 shows the general characteristics of the patients in the two groups. The mean age of the patients in the study group was 27.6 (± 4.6) years while in the control group the mean age was 27.5 (± 4.6) years, and the difference was not statistically significant (t = 0.25, p= 0.80). The mean parity between the two groups were 2.1 (±1.1) and 2.0 (±1.1) and the difference was not statistically significant (t = 0.68, p = 0.50). In the study group (50) 41.0% of the patients were booked while (27) 22.1% were unbooked. In the control group (48) 39.3% of the patients were booked while (26) 21.4% were unbooked. The difference was not statistically significant (X^2^ = 0.16, p = 0.93). The highest educational level for most of the patients in the study group and control group was Quranic education, (58) 47.5% and (57) 46.7% respectively (X^2^ = 0.21, p = 0.98). The mean gestational age at delivery was 39.2 (±1.11) weeks in the study group and 39.4 (± 1.08) weeks in the control group with no statistically significant difference (t = -1.28, p = 0.20). The mean weight on admission was also similar between the two groups, 72.9 (± 7.87) kg in the study group and 73.6 (± 7.64) kg in the control group (t = -0.71, p = 0.48). There were no statistically significant differences in the general characteristics of the patients in both groups.

**Table 1:** general characteristics of patients

Variable	Group A n = 122	Group B n = 122	Test	P-value
Mean age ± SD (years)	27.6 ± 4.6	27.5 + 4.6	t = 0.25	0.80
Mean parity ± SD	2.1 ± 1.1	2.0 + 1.1	t = 0.68	0.50
Mean gestational age at delivery (weeks) ± SD	39.2 ± 1.1	39.4 ± 1.1	t = -1.28	0.20
Mean Weight (kg) ± SD	72.9 ± 7.9	73.6 ± 7.6	t = -0.71	0.48
**Booking status**				
Booked	(50) 41.0%	(48) 39.3%		
Booked elsewhere	(45) 36.9%	(48) 39.3%	X^2^	0.93
Unbooked	(27) 22.1%	(26) 21.4%		
**Educational status**				
Quranic education	(58) 47.5%	(57) 46.7%		
Primary education	(33) 27.1%	(36) 29.5%		
Secondary education	(19) 15.6%	(18) 14.8%	X^2^	0.98
Tertiary education	(12) 9.8%	(11) 9.0%		

Table 2 shows the main indications for caesarean section in the two groups was cephalopelvic disproportion (61) 50.0% and (63) 51.6% in the study and control groups respectively. There was no statistically significant difference in the indications for the operation between the two groups (X^2^ = 0.60, p = 1.0). Regarding the method of lower uterine segment extension, (116) 95.1% and (6) 4.9% of the study group had blunt extension and sharp extension respectively. This was statistically similar to the control group where (120) 98.4% and (2) 1.6% had blunt extension and sharp extension of the lower uterine segment respectively (X^2^ = 2.06, p = 0.15). The placenta was delivered by controlled cord traction in (117) 95.9% and by manual delivery in (5) 4.1% of the patients in the study group. This was statistically similar to the control group, (119) 97.5% and (3) 2.5% respectively (X^2^ = 0.52, p = 0.47). The mean birth weight was 3.3 (± 0.41) kg in the study group and 3.4 (± 0.35) kg in the control group. The difference was not statistically significant (t = -1.71, p = 0.09). The mean duration of the operation was 52.6 (± 5.32) minutes in the study group and 52.5 (± 5.57) minutes in the control group, and the difference was not statistically significant (t = 0.06, p = 0.95). There were no statistically significant differences in the operative characteristics of the patients between the two groups.

**Table 2:** operative characteristics of patients

Variable	Group A n = 122	Group B n = 122	Test	P-value
Indication for operation				
CPD	50.0% (61)	51.6% (63)		
Obstructed labor	12.3% (15)	13.9% (17)		
Malpresentation	11.5% (14)	9.8% (12)		
Fetal distress	9.0% (11)	9.8% (12)	X^2^	1.00
Failed induction of labor	6.6% (8)	5.8% (7)		
Bad obstetrics history in labour	6.6% (8)	5.7% (7)		
Prolonged infertility	4.0% (5)	3.3% (4)		
**Lower uterine extension**				
Blunt	95.1% (116)	98.4% (120)		
Sharp	4.9% (6)	1.6% (2)	X^2^	0.15
**Placental delivery**				
Controlled cord traction	95.9% (117)	97.5% (119)		
Manual	(5) 4.1%	(3) 2.5%	X^2^	0.47
**Mean birth weight (kg)**	3.3 + 0.4	3.4 + 0.4	t = -1.71	0.09
**Mean duration of surgery** (minutes) + SD	52.6 + 5.3	52.5 + 5.6	t = 0.06	0.95
**Mean total blood loss (ml)** + SD	414.0 + 143.7	773.8 + 199.2	t = -16.18	<0.01*

Table 3 shows the comparison of intraoperative blood loss in excess of 1000ml between the two groups. In the study group, intraoperative blood loss was in excess of 1000ml in 2 (1.6%) patients while 120 (98.4%) patients had intraoperative blood loss less than or equal to 1000ml. In the control group, intraoperative blood loss was in excess of 1000ml in 12 (9.8%) patients while 110 (90.2%) patients had intraoperative blood loss less than or equal to 1000ml. There was statistically significant difference in the intraoperative blood loss in excess of 1000ml between the two groups (X^2^ = 7.58, df = 1, p = 0.01). Significantly less number of patients in the study group had intraoperative blood loss in excess of 1000ml. Table 4 shows the changes in haemoglobin and haematocrit levels in the patients in the two groups. The mean preoperative haemoglobin was 10.8 (± 0.8) g/dl in the study group and 10.9 (± 0.6) g/dl in the control group. The difference of the mean was not statistically significant (t = -1.68, p=0.09). The mean postoperative haemoglobin was 10.1 (±0.8) g/dl in the study group and 9.5 (± 0.8) g/dl in the control group. The difference of the mean was statistically significant (t = 4.99, p ≤ 0.01). The mean drop in haemoglobin and percentage drop in haemoglobin in the study group were 0.7 (± 0.4) g/dl and 6.7 (± 3.4) % respectively. The mean drop in haemoglobin and percentage drop in haemoglobin in the control group were 1.4 (± 0.6) g/dl and 12.9 (± 5.4) % respectively. There was statistically significant difference in the mean drop in haemoglobin (t = -10.52, p ≤ 0.01) and percentage drop in haemoglobin (t =- 10.62, p ≤ 0.01) between the two groups.

**Table 3:** comparison of intraoperative blood loss in excess of 1000ml between the two groups

Blood Loss	Group A	Group B	Total
( < 1000)	120 (98.4%)	110 (90.2%)	230
( > 1001)	2 (1.6%)	12 (9.8%)	14
TOTAL	122	122	244

X^2^ = 7.58, df = 1, p =0.01

**Table 4:** changes in haemoglobin and haematocrit levels in the patients

Variables	Group A	Group B	Test	P-value
Mean Pre op# Hb (g/dl) +SD	10.8 +0.8	10.9 + 0.6	t = - 1.68	0.09
Mean Post-op Hb (g/dl) + SD	10.1 + 0.8	9.5+ 0.8	t = 4.99	<0.01*
Mean Drop in Hb (g/dl) + SD	0.7 + 0.4	1.4+ 0.6	t = -10.52	<0.01*
Mean Percent drop in Hb (%) + SD	6.7 + 3.4	12.9 + 5.4	t = -10.62	<0.01*
Mean Pre-op PCV (%) + SD	33.9 + 2.8	34.5 + 2.1	t = -1.78	0.08
Mean Post-op PCV (%) + SD	31.5 + 2.8	29.9 + 2.6	t = 4.70	<0.01*
Mean Drop in PCV (%) + SD	2.3 + 1.1	4.6 + 1.9	t = -11.49	<0.01*
Mean Percent drop in PCV (%) + SD	6.8 + 3.0	13.2 + 5.4	t = - 11.58	<0.01*

* Significant; #operative

The mean preoperative packed cell volume was 33.9 (± 2.8) % in the study group and 34.5 (± 2.1) % in the control group. The difference of the mean was not statistically significant (t = -1.78, p = 0.08). The mean postoperative packed cell volume was 31.5 (± 2.88) % in the study group and 29.9 (±2.6) % in the control group. The difference of the mean was statistically significant (t = 4.70, p ≤ 0.01). The mean drop in packed cell volume and percentage drop in packed cell volume in the study group were 2.3 (±1.1) % and 6.8 (±3.0) % respectively. The mean drop in the packed cell volume and percentage drop in packed cell volume in the control group were 4.6 (±1.9) % and 13.2 (±5.4) % respectively. There was statistically significant difference in the mean drop in packed cell volume (t = -11.49, p ≤ 0.01) and percentage drop in packed cell volume (t = - 11.58, p ≤ 0.01) between the two groups. The mean preoperative haemoglobin and haematocrit levels were statistically similar between the two groups while there was significant reduction in the haemoglobin and haematocrit levels in the control group postoperatively.

Table 5 shows the postoperative complications in the patients in the two groups. Postpartum anaemia was diagnosed in 17 (13.9%) patients in the study group and 31 (25.4%) patients in the control group. Postpartum anaemia was significantly lower in the study group (X^2^ = 5.08, p=0.04). Postpartum haemorrhage was diagnosed in 2 (1.6%) patients in the study group and 12 (9.8%) patients had postpartum haemorrhage in the control group. Postpartum haemorrhage is statistically significant between the two groups (X^2^ = 7.58, p =0.01). Wound infection was diagnosed in 2 (1.6%) patients in the study group and 6 (4.9%) patients in the control group. There was no statistically significant difference in occurrence of wound infection in the two groups (X^2^ = 2.07, p = 0.28). 5(4.1%) patients had blood transfusion in the control group, while none of the patients in study group was transfused with blood. Duration of hospital stay was prolonged in 2 (1.6%) patients in the study group and in 11 (9.0%) patients in the control group. Duration of hospital stay was statistically significant between the two groups (X^2^ = 6.58, p = 0.02).

**Table 5:** postoperative complications in the patients

Complication	Group A (n = 122)	Group B (n = 122)	Test	P-value
			X^2^	
Postpartum Anaemia	17 (13.9%)	31 (25.4%)	5.08	0.04*
PPH	2 (1.6%)	12 (9.8%)	7.58	0.01*
Wound Infection	2 (1.6%)	6 (4.9%)	2.07	0.28
Prolonged Hospital Stay	2 (1.6%)	11 (9.0%)	6.58	0.02*

* Significant

## Discussion

There were no significant differences between the two groups in terms of their general characteristics, labour characteristics, pre-operative haematocrit and haemoglobin values and operative characteristics in this study. This shows that these variables, which could affect the amount of blood loss during the surgery, were similar in the two groups and the observed differences in the intraoperative blood loss, was mainly due to the administration of the preoperative dose of tranexamic acid as an adjunct to oxytocin in reducing blood loss during caesarean section in one group since all the patients in both groups received oxytocin after the delivery of the neonate according to protocol.

In this study (Table 2), the average blood loss during caesarean section when preoperative tranexamic acid was not given was found to be relatively high (773 ± 199) ml. This is similar to the findings from a study in Karachi (787±519) ml where blood loss at both elective and emergency caesarean section was calculated based on patients´ blood volume and drop in haematocrit [29]. Also similar to the findings from this study is that from a Randomised controlled trial which found out that average blood loss at caesarean section was 606 ± 193 ml [27]. This result was found in patients who received only oxytocin after delivery of the baby at elective caesarean section without adjunctive administration of tranexamic acid. One study in India also reported an average blood loss of 602 ml, when preoperative tranexamic acid was not administered [3]. The average blood loss at caesarean section when preoperative tranexamic acid was administered as an adjunct to oxytocin in this study was found to be relatively lower (414 ± 143) ml. This is similar to findings in a study in Istanbul, Turkey where intravenous tranexamic acid was administered prior to caesarean section. The blood loss at surgery in the study group was (499±206) ml [30]. This finding is also comparable to a study in India where two different doses of tranexamic acid was compared with a placebo as adjunct to oxytocin, intraoperative blood loss was significantly lower in both of the tranexamic groups (262±31) ml and (376±31) ml respectively compared to the control group (527±88) ml [21]. In this study, the average blood loss at caesarean section when preoperative tranexamic acid was administered as an adjunct to oxytocin in reducing blood loss was found to be significantly lower when compared to oxytocin alone. This finding was similar to other studies where tranexamic acid was compared to placebo as an adjunct to oxytocin [31].

In this study, the proportion of patients who had bleeding following caesarean section in excess of 1000 ml was significantly lower, 1.6% (2) patients in the study group compared with 9.8% (12) patients in the control group. The administration of preoperative tranexamic acid in this study, was associated with 15% less likelihood of developing postpartum haemorrhage [OR= -1.88, AOR= 0.15, p= 0.15, 95% CI= (0.03-0.70)]. This finding is similar to that in a Randomised controlled study where the proportion of women who experienced an estimated blood loss greater than 1000 ml was significantly lower in the tranexamic acid group 2.1% compared to the control group 5.8% [32]. In this study (Table 4), the postoperative haemoglobin and haematocrit were significantly higher in the study group (10.1 ± 0.8 g/dl and 31.5 ± 2.88% respectively) compared to the control group (9.5±0.8g/dl and 29.9 ± 2.6% respectively). Other studies found similar results [21, 26, 27]. One study was conducted in anaemic parturients and preoperative tranexamic acid proved to be a useful drug to prevent blood loss in these particular patients undergoing caesarean section [21]. In this study, there is significantly lesser drop in haemoglobin and haematocrit levels in the study group compared to the control group. This is similar to findings in other studies, where there was significantly lesser drop in haemoglobin and haematocrit in the tranexamic group compared to the control group [26, 32].

In this study, postpartum anaemia was the commonest complication following caesarean section. The incidence of postpartum anaemia was significantly higher in the control group 31 (25.4%) patients. This is comparable to a study on Caesarean morbidity and mortality in our centre which found an incidence of postpartum anaemia of 21.3% as commonest complication following caesarean section [20]. In this study, the incidence of postpartum anaemia was significantly reduced in the study group 17 (13.9%). Other studies found postpartum anaemia to be the highest morbidity following caesarean section [18, 20]. In this study (Table 5), the proportion of patients with prolonged hospital stay was 11 (9.0%) patients in the control group and 2 (1.6%) patients in the study group. The incidence of prolonged hospital stay was statistically significant between the two groups. There is paucity of data and studies comparing the effect of preoperative tranexamic acid as an adjunct to oxytocin on hospital stay with which to further compare the findings in this study. However, the proportion of patients in the control group 9.0% who experienced prolonged hospital stay is similar to an earlier study in our centre that found the incidence of prolonged hospital stay following caesarean section to be 9.4% [20].

## Conclusion

This study found a significant reduction in the blood loss during emergency caesarean section with the administration of tranexamic acid before commencement of caesarean section. There was also a significant reduction in postpartum haemorrhage, postpartum anaemia; the need for blood transfusion and length of hospital stay associated with the administration of the drug. We recommend that tranexamic acid should be given preoperatively as an adjunct to oxytocin for reduction of intra operative blood lost.

### What is known about this topic

- Postpartum haemorrhage (PPH) is the main cause of maternal mortality worldwide especially in developing countries;
- Tranexamic acid is effective for treatment of postpartum haemorrhage.

### What this study adds

- Tranexamic acid is also effective in prevention of blood lost during caesarean section;
- Tranexamic acid reduces the need for blood transfusion and duration of hospital stay if administered before surgery.
